# Comparative analysis of metabolic and functional cardiac alterations in diet‐ and genetically induced mouse models of cardiac dysfunction

**DOI:** 10.1111/febs.70362

**Published:** 2025-12-12

**Authors:** Christiane Ott, Patricia Baumgarten, Thorsten Henning, Daniela Weber, Iwona Wallach, Jana Grune, Ulrich Kintscher, Hermann‐Georg Holzhütter, Tilman Grune, Nikolaus Berndt

**Affiliations:** ^1^ Department of Molecular Toxicology German Institute of Human Nutrition Potsdam‐Rehbruecke (DIfE) Nuthetal Germany; ^2^ DZHK (German Centre for Cardiovascular Research) Berlin Germany; ^3^ Food4Future (F4F) c/o Leibniz Institute of Vegetable and Ornamental Crops (IGZ) Germany; ^4^ Department of Radiology Charité – Universitätsmedizin Berlin, Corporate Member of Freie Universität Berlin and Humboldt‐Universität zu Berlin Germany; ^5^ Department of Cardiothoracic and Vascular Surgery Deutsches Herzzentrum der Charité (DHZC) Berlin Germany; ^6^ Charité – Universitätsmedizin Berlin, Corporate Member of Freie Universität Berlin and Humboldt‐Universität zu Berlin Germany; ^7^ Institute of Physiology, Charité – Universitätsmedizin Berlin, Corporate Member of Freie Universität Berlin and Humboldt‐Universität zu Berlin Germany; ^8^ Institute of Pharmacology, Max Rubner Center for Cardiovascular Metabolic Renal Research, Charité – Universitätsmedizin Berlin, Corporate Member of Freie Universität Berlin and Humboldt‐Universität zu Berlin Germany; ^9^ Institute of Biochemistry, Charité – Universitätsmedizin Berlin, Corporate Member of Freie Universität Berlin and Humboldt‐Universität zu Berlin Germany; ^10^ German Center for Diabetes Research (DZD) München‐Neuherberg Germany

**Keywords:** cardiac dysfunction, cardiac metabolism, genetically induced obesity, high‐fat diet, metabolic adaptation

## Abstract

Cardiac metabolism is highly adaptive, and distinct maladaptive remodeling processes may contribute to the development of cardiac dysfunction. Here, we compared the metabolic, structural, and functional adaptations of two murine models: C57BL/6J mice fed a high‐fat, carbohydrate‐free diet and New Zealand Obese mice maintained on a standard diet. Cardiac function was assessed by echocardiography, plasma metabolite profiles were analyzed, and cardiac proteomes were quantified by mass spectrometry. Proteomic data were computationally integrated into a kinetic model of cardiac central metabolism (CARDIOKIN1) to predict changes in substrate utilization and ATP production capacities under physiological nutrient conditions. Diet‐induced metabolic stress led to cardiac dysfunction with preserved ejection fraction, characterized by mitochondrial dysfunction, impaired ATP production, inflammation, and reduced cardiac mass. Conversely, genetically induced obesity resulted in cardiac impairment with reduced ejection fraction associated with mild fibrosis, maintained ATP production, and substrate switching favoring fatty acid utilization. Proteomic and computational analyses revealed a coordinated downregulation of metabolic networks involved in oxidative phosphorylation, substrate transport, and energy production in both models, but with distinct profiles of metabolic inflexibility and mitochondrial efficiency. This study provides insights of how dietary versus genetic metabolic stress reprograms cardiac metabolism and structure, offering mechanistic insights into the diverse pathways leading to cardiac dysfunction. These insights may guide future strategies for metabolic intervention in heart failure subtypes.

## Graphical abstract

Dietary and genetic stress trigger different routes to heart failure. In mice, a carbohydrate‐free high‐fat diet caused mitochondrial dysfunction and impaired energy production with preserved pumping function, while genetic obesity led to heart enlargement, fibrosis, and reduced function. By integrating proteomics with computational modeling, we show how distinct metabolic shifts drive specific forms of cardiac impairment, offering insights for tailored therapeutic strategies.

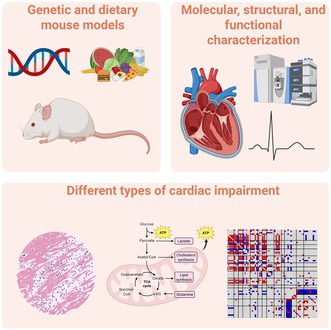

**Abbreviations**
BW: body weight
ATP: adenosine triphosphate
BCAA(s): branched‐chain amino acid(s)
Black6 SD/HF: black 6 mice (C57BL/6JRj) on a standard/high‐fat no‐carbohydrate diet
CO: cardiac output
EDV: end‐diastolic volume
EF: ejection fraction
ESV: end‐systolic volume
HF: high‐fat (no‐carbohydrate) diet
HFpEF: heart failure with preserved ejection fraction
HFrEF: heart failure with reduced ejection fraction
LAGESO: Landesamt für Gesundheit und Soziales
LAVG: Landesamt für Arbeitsschutz Verbraucherschutz und Gesundheit
LV: left ventricle or left ventricular
LVAWs/d: LV anterior wall systole/diastole
LVIDs/d: LV inner diameter systole/diastole
LVM: LV mass
LVPWs/d: LV posterior wall systole/diastole
NEFAs: nonesterified fatty acids
NZO: New Zealand Obese mice
NZO SD: NZO mice on a standard diet
PCA: principal component analysis
SD: standard diet
SV: stroke volume
UCPs: uncoupling proteins

## Introduction

Nowadays, heart failure is classified into two primary phenotypes: heart failure with preserved ejection fraction (HFpEF) and heart failure with reduced ejection fraction (HFrEF). These phenotypes exhibit distinct clinical characteristics, underlying mechanisms, and responses to treatment [1, 2, 3]. HFpEF is characterized by diastolic dysfunction where the heart's ability to relax and fill with blood is impaired [4, 5, 6]. In contrast, HFrEF involves systolic dysfunction where the heart's ability to contract and pump blood is compromised [7].

Beyond structural and functional changes, metabolism plays a pivotal role in heart failure, as disruptions in metabolic processes can significantly impair cardiac function [8, 9]. The heart is one of the most energetically demanding organs, with one‐third of the cellular volume of cardiac myocytes occupied by mitochondria [10]. This high mitochondrial content supports the heart's substantial energy requirements. Per gram of tissue, the heart has the highest oxygen consumption rate, and the daily utilization of adenosine triphosphate (ATP) amounts to 20 times its weight. Maintaining cardiac functionality requires a robust and continuous generation of ATP, necessary for both electrophysiological processes, such as ion pumping, and the mechanical work of the contractile apparatus.

To ensure a sufficient ATP supply, the heart utilizes various energy sources, including carbohydrates, amino acids, lipids, and ketone bodies. Under normal conditions, the heart primarily relies on the oxidation of free fatty acids, which contribute approximately 70% of its ATP production [11]. However, the proportion of different energy‐providing substrates can vary with physiological and pathophysiological conditions (e.g., starvation, systemic inflammation, high physical stress) and dietary compositions (relative proportions of fat, protein, and carbohydrate).

Disruptions in metabolic processes can lead to impaired ATP production, directly affecting the heart's contractile function and overall performance. Recent advances in cardiac metabolism research have highlighted the significant role of metabolic alterations in heart failure [8, 9]. The failing heart often undergoes distinct metabolic adaptations in HFpEF and HFrEF. In HFrEF, there is a notable reduction in fatty acid oxidation with a compensatory increase in glucose utilization. In contrast, HFpEF shows a more variable metabolic profile, with some studies reporting preserved or even increased fatty acid oxidation, while others indicate increased reliance on glycolysis under certain conditions. This suggests that metabolic shifts in HFpEF are more heterogeneous and context‐dependent compared to HFrEF [2, 3]. Since the specific metabolic profiles and underlying molecular mechanisms can vary significantly, there is a need for a better understanding to develop targeted interventions and therapeutic strategies aimed at modulating cardiac energy metabolism.

The influence of nutrition on cardiac performance is becoming increasingly important due to dietary changes characterizing the ‘nutrition transition’ in industrialized countries. These changes include shifts towards higher energy density diets with more fat and added sugars, greater saturated fat intake, and reduced intakes of complex carbohydrates and dietary fiber [12]. This ‘nutrient transition’ is closely associated with the increasing prevalence of obesity, type 2 diabetes, musculoskeletal disorders, and cardiovascular diseases, making metabolic syndrome highly prevalent in patients with heart failure [13]. While there are clear links between dietary habits, obesity, and heart failure, the underlying molecular adaptations and associated metabolic alterations are still not fully understood and may be heterogeneous depending on diet and genetic predisposition.

Animal models are essential for studying metabolic adaptations in heart failure, providing insights into the mechanisms driving these conditions. This study hypothesizes that distinct metabolic stress conditions induced by diet or genetic predisposition to obesity underpin the development of different phenotypes cardiac dysfunction. Specifically, we expect that black 6 mice (C57BL/6JRj) on only a high‐fat diet (Black6 HF) will exhibit metabolic inflexibility, characterized by impaired glucose utilization and reliance on fatty acids, leading to cardiac impairment. In contrast, New Zealand Obese mice on a standard diet (NZO SD), which are genetically predisposed to obesity, diabetes, and impaired cardiac function [14], are assumed to shift towards glucose metabolism with maintained ATP production capacity, reflecting a HFrEF‐like phenotype. This hypothesis is grounded in the existing literature that reports differential metabolic adaptations in HFpEF and HFrEF [2, 3]. To characterize the NZO SD and Black6 HF mouse models for their cardiometabolic adaptations and development of cardiac dysfunction, we combined functional cardiac imaging through echocardiography with analysis of plasma metabolites and tissue proteomics to investigate the underlying molecular and metabolic mechanisms of the different phenotypes. Using a comprehensive mathematical model of myocardial metabolism [15], we identify metabolic adaptations underlying decreased cardiac function. Our analysis reveals clear differences in cardiometabolic characteristics reflecting functional and structural adaptations in the two distinct models of metabolic stress, whereby male NZO SD mice showed cardiac dysfunction with reduced EF phenotype and male Black6 HF mice unexpectedly displayed cardiac impairment with a maintained EF.

This study enhances our understanding of the metabolic underpinnings of cardiac dysfunction and underscores the importance of considering metabolic phenotypes.

## Results

### High‐fat diet and NZO strain both lead to impaired cardiac function

We aimed to analyze metabolic and functional cardiac alterations in the heart of two different mouse models of metabolic stress with regard to the development of cardiac dysfunction. On one hand, we used black 6 mice receiving a high‐fat, no‐carbohydrate diet for 13 weeks (Black6 HF) mimicking metabolic inflexibility in the development of cardiac impairment through diet. On the other hand, we used the NZO strain, a polygenic model for metabolic syndrome that spontaneously developed obesity [16], degenerative aortic valve disease, and cardiac dysfunction [14] on a standard diet (NZO SD). As a control group, we used black 6 mice receiving a standard diet (Black6 SD).

**Fig. 1:**
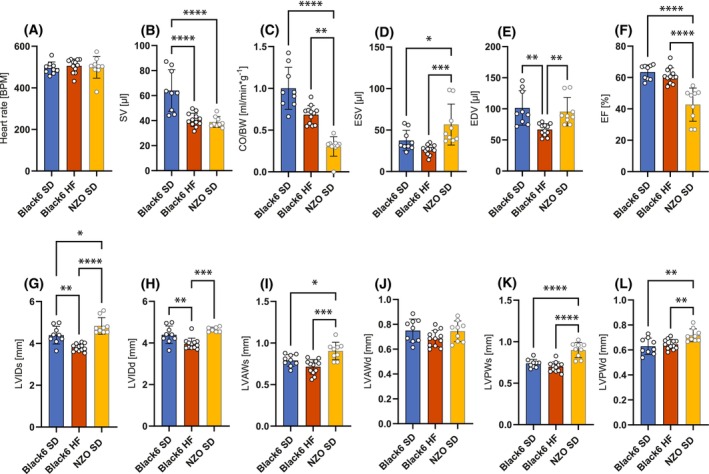
Echocardiographic parameters of left ventricular (LV) function in male Black6 SD (black 6 mice on a standard diet; *n* = 9), Black6 HF (black 6 mice on a high‐fat no‐carbohydrate diet; *n* = 12), and NZO SD mice (New Zealand Obese mice on a standard diet; *n* = 9). (A) Heart rate. BPM, beats per minute; (B) SV, stroke volume; (C) CO/BW, cardiac output per body weight; (D) ESV, end‐systolic volume; (E) EDV, end‐diastolic volume; (F) EF, ejection fraction; (G, H) LVIDs/d, LV inner diameter systole/diastole; (I, J) LVAWs/d, LV anterior wall systole/diastole; (K, L) LVPWs/d, LV posterior wall systole/diastole. Bars represent mean ± SD, and individual points correspond to biological replicates. Data were tested for normality using the Shapiro–Wilk or Kolmogorov–Smirnov test. Statistical significance was determined by one‐way ANOVA followed by Tukey's multiple comparison test. *P < 0.05, **P < 0.01, ***P < 0.001, ****P < 0.0001.

To characterize and compare the functional cardiac phenotype, we performed echocardiography investigating geometric and functional parameters including heart rate, stroke volume (SV), cardiac output relative to body weight (CO/BW), end‐systolic (ESV) and end‐diastolic volume (EDV), EF, LV internal diameter at end‐systole (LVIDs) and end‐diastole (LVIDd), LV anterolateral wall thickness at end‐systole (LVAWs) and end‐diastole (LVAWd), as well as LV posterior wall thickness at end‐systole (LVPWs) and end‐diastole (LVPWd). While heart rate was not significantly different between the three groups (Fig. 1A), SV was significantly decreased by about one‐third in Black6 HF and NZO SD mice compared to Black6 SD mice (Fig. 1B). CO/BW was severely decreased in NZO SD mice, but not in Black6 HF mice (Fig. 1C), although there was a trend (*P*‐value = 0.087). ESV was significantly increased in the NZO SD mice only, but remained normal in Black6 HF mice (Fig. 1D), while EDV was decreased in the Black6 HF mice, but remained normal in the NZO SD group (Fig. 1E). EF was around 60% in Black6 SD and Black6 HF mice but was severely reduced to around 40% in NZO SD mice (Fig. 1F). Besides functional differences, we also found significant differences in the geometric parameters of the heart. LVIDs and LVIDd were significantly reduced in the Black6 HF mice, but not in the NZO SD mice, where LVIDd was significantly increased (Fig. 1G,H). LVAWs was significantly increased in the NZO SD group, but not in the Black6 HF group (Fig. 1I). There was no significant difference in LVAWd (Fig. 1J). LVPWs and LVPWd were significantly increased in the NZO SD mice but remained normal in the Black6 HF group (Fig. 1K,L). Although both Black6 HF and NZO SD mice developed profound cardiac dysfunction, marked differences in EF were observed, with Black6 HF mice displaying a mean EF of 61.2% and NZO SD mice displaying a mean EF of 42.7%, suggesting a differential development of cardiac impairment through the metabolic challenges.

### Cardiac function relies on changes in cardiac mass and is triggered by plasma metabolic changes in NZO SD but not in Black6 HF animals

**Fig. 2:**
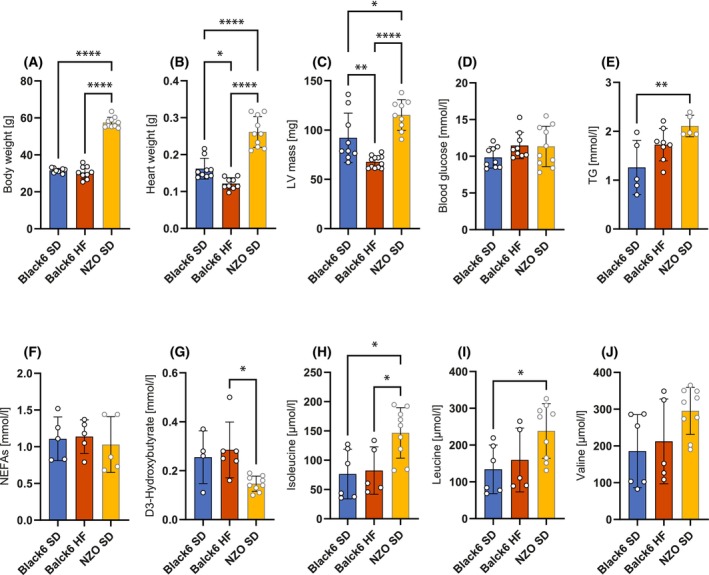
Physiological parameters and plasma metabolites. (A, B) Body and heart weight (*n* = 9–10); (C) LV mass (*n* = 9–12); (D) Blood glucose (*n* = 9–10); (E) TG, triglycerides (*n* = 5–8); (F) NEFAs, nonesterified fatty acids (*n* = 5). (G) D‐3‐hydroxybutyrate (*n* = 4–9); (H–J) branched‐chain amino acids (BCAAs) (*n* = 5–9). Bars represent mean ± SD, and individual points correspond to biological replicates. Data were tested for normality using the Shapiro–Wilk or Kolmogorov–Smirnov test. Statistical significance was determined by one‐way ANOVA followed by Tukey's multiple comparison test. *P < 0.05, **P < 0.01, ****P < 0.0001.

We monitored body and heart muscle mass as well as LV mass. In comparison to controls, Black6 HF mice showed no differences in body mass after 13 weeks, but significantly reduced heart and LV mass (Fig. 2A–C). This was interesting because it disconfirms the current belief that loss of cardiac function is always directly linked to increased body and heart mass in high‐fat diet mouse models.

To better understand the relationship between cardiac function and diet, we monitored changes in key plasma metabolites including glucose, triglycerides (TG), NEFAs, hydroxybutyrate, and BCAAs (leucine, isoleucine, and valine). The Black6 HF mice exhibited no noteworthy alterations in the primary energy‐delivering substrates, such as glucose, NEFAs, and TG (Fig. 2D–F), as well as alternative substrates, such as ketone bodies and BCAAs (Fig. 2G–J). This is in stark contrast to the NZO SD mice, which showed a significant increase in body weight, cardiac mass, and LV mass compared to Black6 SD mice (Fig. 2A–C), which were accompanied by significant alterations in circulating nutrients, displaying increased levels of circulating TG (Fig. 2E), leucine, and isoleucine (Fig. 2H,I), and decreased levels of hydroxybutyrate (Fig. 2G), while glucose and NEFA levels remained unchanged (Fig. 2D,F). These findings suggest that circulating plasma nutrients contribute to diminished cardiac function in NZO SD mice but not in Black6 HF mice. Importantly, the opposite development of cardiac and LV mass shows that deteriorating cardiac function in Black6 HF mice goes along with diminished cardiac tissue, while NZO SD mice have a compensatory mechanism leading to hypertrophy and increased cardiac muscle mass.

### Overall proteomic changes

**Fig. 3:**
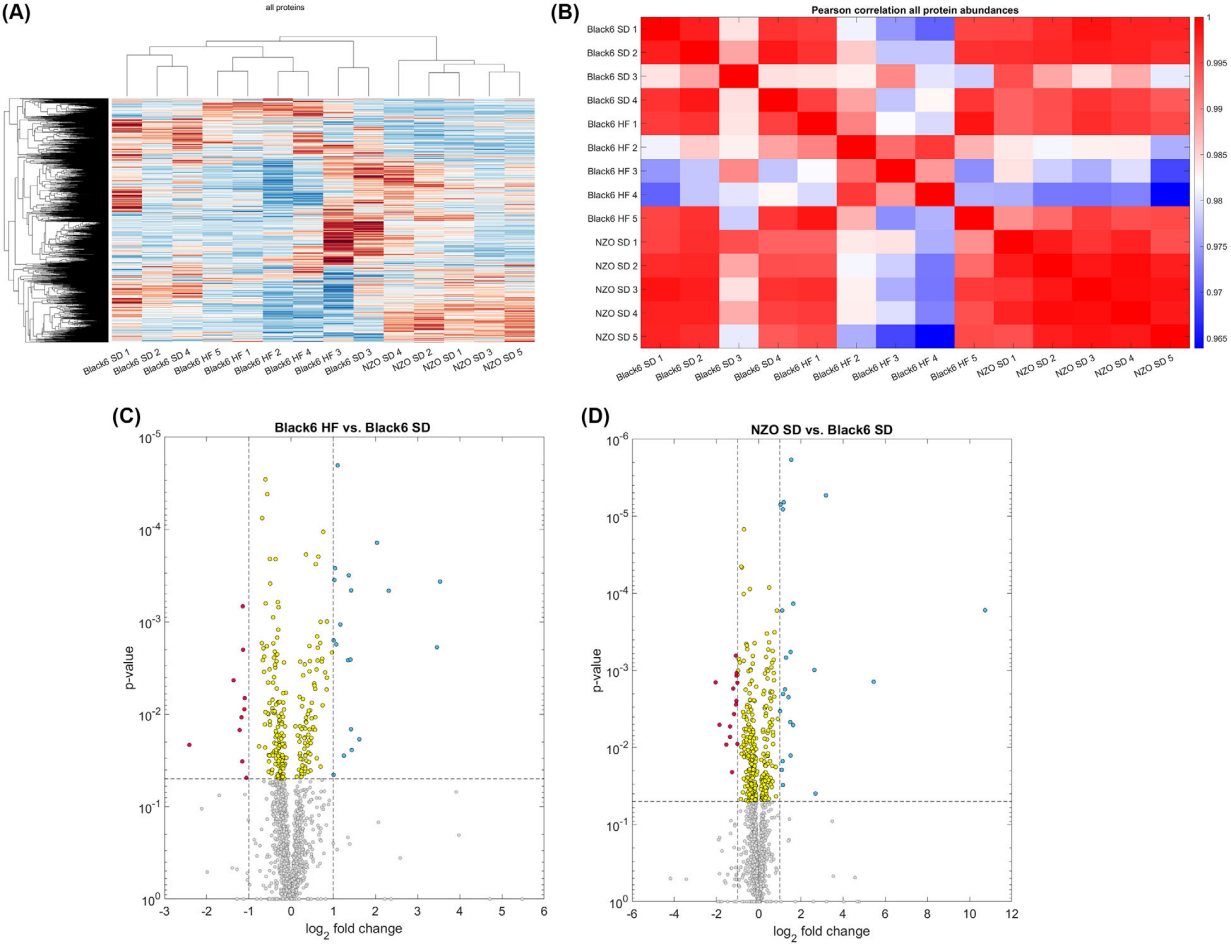
Protein abundance changes. (A) Clustering of the whole proteomic samples. DIA‐NN (Data‐Independent Acquisition–Neural Network) mass spectrometry data were analyzed by nonsupervised hierarchical clustering and z‐score normalization. (B) Pearson correlation between individual samples for all proteins. (C + D) Volcano plots showing the log_2_ fold changes of all proteins detected in Black6 HF mice (*n* = 5) (C) and NZO SD mice (*n* = 5) (D) compared to the Black6 SD mice (*n* = 4). The left side corresponds to proteins that are downregulated in Black6 HF, while the right side corresponds to proteins that are upregulated. Red dots represent significantly downregulated proteins with a fold change < 0.5, while blue dots represent significantly upregulated proteins with a fold change > 2. The significance of the fold changes could be estimated for 2470 proteins.

To better understand the underlying molecular and proteomic changes, we determined the cardiac proteome by mass spectrometry in Black6 SD, Black6 HF, and NZO SD mice. DIA‐NN mass spectrometry data were analyzed by nonsupervised hierarchical clustering and *z*‐scoring to generate heatmaps containing 2470 proteins within multiple clusters (Fig. 3A). Overall, individual protein intensity profiles showed a high correlation (*R* > 0.95) (Fig. 3B). The volcano plots in Fig. 3C,D illustrate differences between the mean protein intensities of Black6 HF and NZO SD groups in comparison to the Black6 SD group.

### Changes in cardiac energy metabolism

**Fig. 4:**
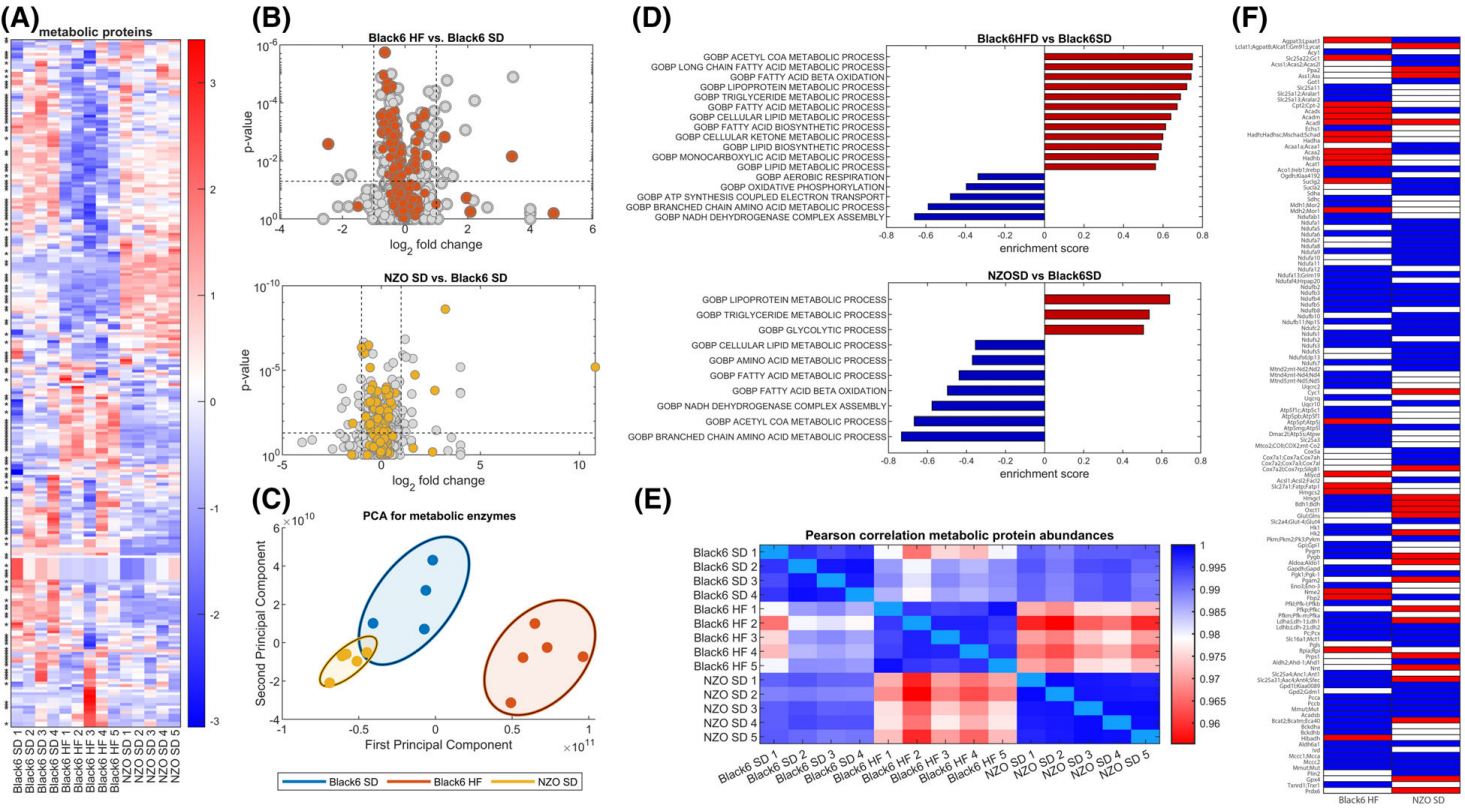
Cardiometabolic changes at the protein level based on proteomic data. (A) *Z*‐score normalized unbiased cluster analysis of metabolic enzymes identifies distinct clusters of proteins differentially regulated between the different groups. (B) Volcano plots showing the log_2_ fold changes of all proteins detected in Black6 HF mice (*n* = 5; upper panel) and NZO SD mice (*n* = 5; lower panel). The subset of metabolic proteins is highlighted in orange and yellow, respectively. The left side corresponds to proteins that are downregulated, while the right side corresponds to proteins that are upregulated. The significance of the fold changes could be estimated for 2470 proteins. While only about 15% of all proteins are significantly altered in both cardiac dysfunction models, a fraction of significantly regulated metabolic proteins comprises > 30%. (C) Principal component analysis (PCA) of metabolic enzymes indicates a clear separation of the groups. Correlation between individual samples for all proteins. (D) Gene set enrichment analysis revealed significantly enriched metabolic functions for the two animal models. GOBP, Gene Ontology – Biological Process; (E) Pearson correlation between individual samples for metabolic proteins. (F) Significantly altered metabolic proteins between Black6 HF as well as NZO SD and the control group. Blue indicates significant downregulation (*P* < 0.05) and red indicates significant upregulation (*P* > 0.05).

Metabolic alterations are a key component of heart failure [8, 9], and we demonstrated recently that metabolic alterations play a crucial role in various heart failure models [17, 18, 19] as well as in human aortic stenosis and mitral valve disease [15]. The alterations in plasma nutrient composition (Fig. 2D–J) hint at a metabolic adaption on the whole‐body level that might also directly influence the cardiac ability to utilize different sources of energy‐delivering substrates and potentially also alter the cardiac ability to generate the energy needed for proper cardiac functionality. Hierarchical clustering of the subset of metabolic enzymes identified different clusters differentially regulated between control and cardiac dysfunction models (Fig. 4A). Inspection of volcano plots highlighting the subset of metabolic enzymes (Fig. 4B) revealed downregulation of metabolic enzymes in Black6 HF as well as NZO SD. Principal component analysis demonstrated a clear separation between the groups along the first principal component, with a more pronounced distinction for Black6 HF than for NZO compared with Black6 SD mice (Fig. 4C).

Gene set enrichment analysis using the GO term database (Fig. 4D) revealed a significant upregulation of fatty acid metabolism in Black6 HF animals, together with a downregulation of key components of mitochondrial energy production and BCAA metabolism. In contrast, gene set enrichment analysis indicated increased glycolysis and partially conflicting regulation of fatty acid metabolism in NZO SD mice.

The group differences observed in the PCA were further supported by the overall similarity structure of metabolic protein abundances shown in the Pearson correlation heatmap (Fig. 4E). Just looking at significantly regulated metabolic enzymes (Fig. 4F) also indicated a predominant downregulation of metabolic processes in both Black6 HF and NZO SD mice. Overall, 42% of metabolic enzymes in Black6 HF mice were significantly altered (7.4% upregulated, 34.6% downregulated), a much higher fraction compared to the 15.8% of regulated proteins in the whole proteome (3.2% upregulated, 12.5% downregulated). Similarly, in NZO SD mice, 33.5% of metabolic proteins were significantly regulated (10.7% upregulated, 22.8% downregulated), compared to 13.5% in the whole proteome (6.5% upregulated, 7% downregulated). This finding suggests a critical role for metabolic adaptation in cardiac dysfunction, with metabolic processes being particularly responsive and possibly more affected under the conditions studied.

### Metabolic flexibility

To better understand the physiological relevance of the observed proteomic changes in metabolic enzymes, we integrated protein abundance data of metabolic enzymes into CARDIOKIN1, a molecular resolved kinetic model of central cardiac metabolism [15]. As metabolic flexibility, that is, the ability to use various energy‐delivering substrates, is one of the most important metabolic characteristics of the heart [20] enabling the maintenance of cardiac functionality regardless of dietary conditions, we first investigated the heart‐specific capacities for the utilization of glucose, lactate, fatty acids (NEFAs), BCAAs (valine, leucine, isoleucine), and ketone bodies (β‐hydroxybutyrate, acetoacetate) as described by Berndt *et al*. [15].

**Fig. 5:**
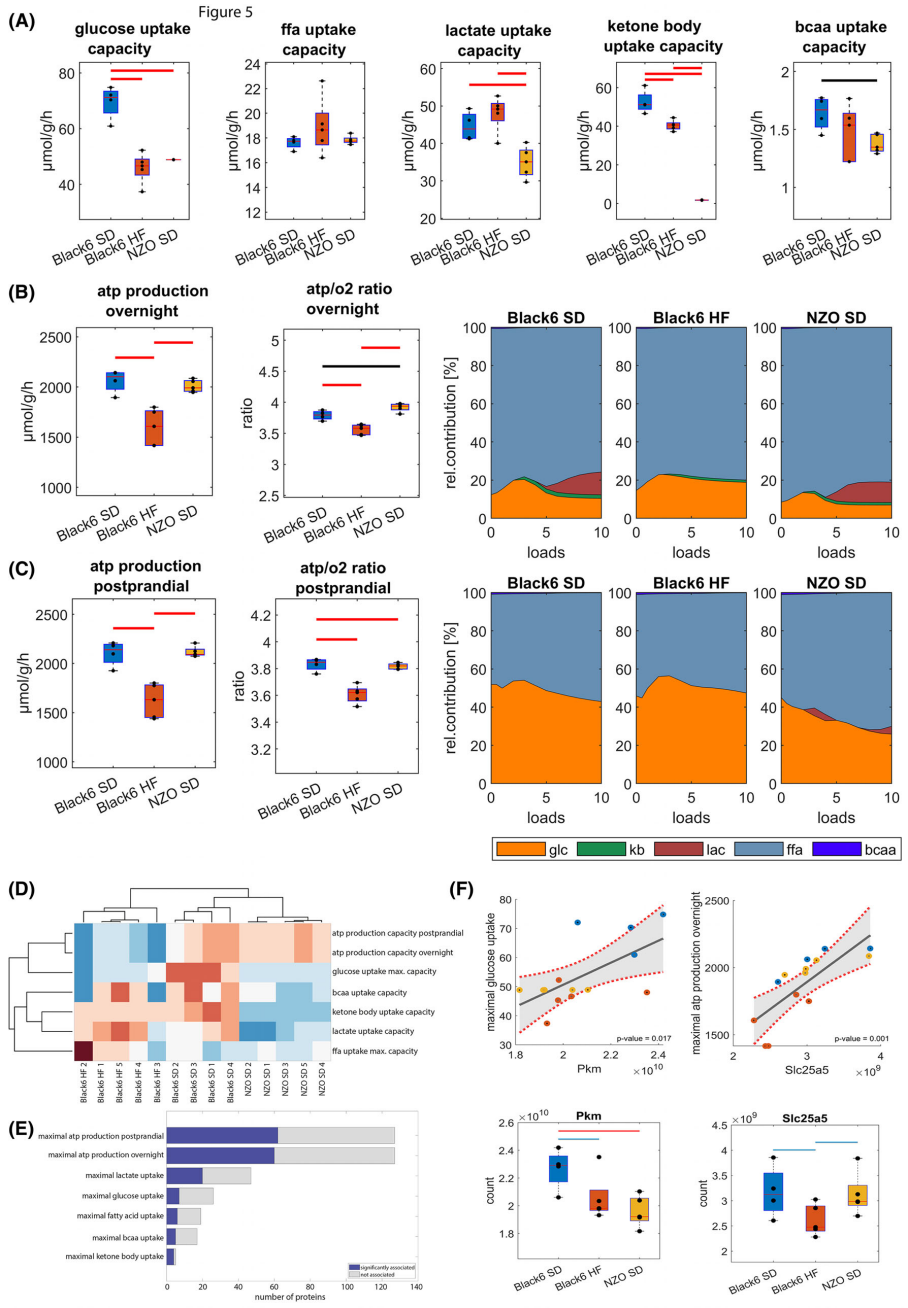
Cardiometabolic changes in utilization capacities for energy‐delivering substrates assessed by computational modeling. Sample sizes: Black6 SD (*n* = 4), Black6 HF (*n* = 5), and NZO SD (*n* = 5) for all panels. (A) Maximal substrate utilization rates: glucose, free fatty acids (ffa), lactate, ketone bodies, and branched‐chain amino acids (bcaa). Differences are shown between the control group (Black6 SD) and Black6 HF mice or NZO SD mice. (B + C) Maximal ATP production capacity and ATP/O_2_ ratio (left panels) under overnight fasted conditions (B) and postprandial conditions (C); the relative contribution of energy‐delivering substrates to total energy expenditure at different energetic demands (expressed as group means of ATP equivalents; right panels); glc, glucose; kb, ketone bodies; lac, lactate; (D) Unbiased clustering of metabolic capacities and maximal ATP production capacities for all individual samples. (E) The number of metabolic proteins significantly associated with the capacity of their respective pathway (blue) and the total number of proteins belonging to the pathway (gray). (F) Two examples of a correlation between metabolic function and individual proteins and the distribution of protein abundance between the different groups. Top: The black line indicates the linear regression fit, with the shaded gray area denoting the 95% confidence interval. Data points represent individual samples, color‐coded by group: Black6 SD (blue), Black6 HF (red), and NZO SD (yellow). Box plots in (A, B, C and F) indicate the median and interquartile range (25th–75th percentiles), with whiskers representing data variability and individual data points shown as black dots. Group values were checked for normality by a one‐sample Kolmogorov–Smirnov test. Statistical group differences were calculated by unpaired two‐sided Student's t‐test for normally distributed group values; otherwise, the Wilcoxon signed‐ranked test was used. Colored horizontal lines indicate statistically significant differences between groups (blue, *P* < 0.1; black; *P* < 0.05; red, *P* < 0.01).

Figure 5A shows a strong reduction in cardiac uptake capacity for glucose in the Black6 HF and NZO SD groups, but no changes in fatty acid uptake capacity between the three groups, although this might be blurred by the large scattering in the Black6 HF group. Lactate uptake capacity was unchanged in the Black6 HF group and significantly decreased in the NZO SD mice, and ketone body uptake capacity was found to be significantly reduced in the Black6 HF group and almost absent in NZO SD mice. BCAA utilization was unchanged in the Black6 HF group and significantly reduced in NZO SD mice, despite an increase in BCAA plasma levels. Overall, this indicates diminished metabolic flexibility in both Black6 HF and NZO SD mice.

Reduced metabolic flexibility and altered capacities for substrate utilization might contribute to overall reduced ATP production capacity in the heart [9, 20]. We defined the ATP production capacity of the heart as the maximal ATP production rate that can be sustained by the individual hearts at given blood plasma nutrient compositions. To cover the range of physiological dietary conditions, we used two different metabolic states, a fasted state defined by high concentrations of fatty acids and a decreased availability of glucose, and a postprandial state characterized by high levels of glucose and decreased fatty acid and lactate availability. As shown in Fig. 5B,C (left panels), we found a clear reduction in the maximal ATP production capacities of the Black6 HF group, but not in the NZO SD group for both overnight fasted and postprandial conditions. The decrease in ATP production capacity goes along with a decrease in mitochondrial efficiency as measured by the ATP/O_2_ ratio. Across all energy demands (‘loads’), fatty acids are the main energy‐delivering substrate contributing about 80% to the overall ATP production under overnight fasted conditions (Fig. 5B, right panels). At high‐energy demand (loads > 5), lactate contributes to the overall ATP production in Black6 SD and NZO SD mice, but not in Black6 HF mice. Under postprandial conditions (Fig. 5C, right panels), the relative share of fatty acids decreases to ~50% in Black6 SD and Black6 HF mice but is still at ~70% in NZO SD mice at high‐energy demand.

**Fig. 6:**
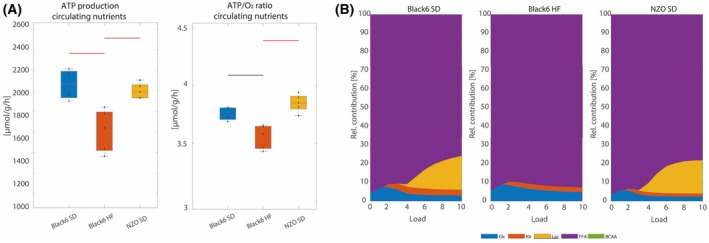
Maximal ATP production and substrate contributions under individualized metabolic conditions. Sample sizes: Black6 SD (*n* = 4), Black6 HF (*n* = 5), and NZO SD (*n* = 5) for all panels. (A) The maximal ATP production capacity and ATP/O_2_ ratio under metabolic conditions corresponding to individual plasma nutrient profiles (see Fig. 2). Box plots indicate the median and interquartile range (25th–75th percentiles), with whiskers representing data variability and individual data points (maximal capacities for individual animals) shown as black dots. Group values were checked for normality by a one‐sample Kolmogorov–Smirnov test. Statistical group differences were calculated by unpaired two‐sided Student's *t*‐test for normally distributed group values; otherwise, the Wilcoxon signed‐ranked test was used. Colored horizontal lines indicate statistically significant differences between groups (black; *P* < 0.05; red, *P* < 0.01). (B) The contribution of energy‐delivering substrates to total energy expenditure at varying energetic demands, expressed as group means of ATP equivalents, within these individualized conditions.

The ATP production capacity was assessed under identical fixed metabolic conditions to isolate the impact of proteomic‐level adaptations. To account for the potential influence of nutrient availability on cardiac metabolic function, we also performed simulations incorporating changes in circulating plasma nutrients as shown in Fig. 2. However, since the observed changes in plasma nutrients were limited to ketone bodies and branched‐chain amino acids—substrates that contribute minimally to overall energy production—no significant modifications of ATP production capacity (Fig. 6A) and ATP/O_2_ ratio (Fig. 6B) were detected.

Considering the changes in cardiac mass (Fig. 2B,C), there is an increase in the maximal ATP production capacity for the whole heart in NZO SD mice and an even more pronounced decrease in the Black6 HF group. This indicates that the Black6 HF group is characterized by diminished metabolic capacity contributing to reduced cardiac function, while the hearts of the NZO SD group compensate for the functional loss of heart tissue through an increase in cardiac mass. Overall, the metabolic adaptations allow a clear separation of the three groups by a *z*‐score normalized unbiased clustering (Fig. 5D).

As metabolic alterations, especially in glucose metabolism and ATP production capacity, are key determinants of cardiac dysfunction in Black6 SD as well as NZO mice, we aimed to identify functional molecular markers that have the potential to predict metabolic function in individual samples. We used linear regression models to assess the association of maximal metabolic capacities with the abundance of proteins belonging to the respective pathway. Overall, we identified 7 out of 26 glycolytic enzymes, 6 out of 19 enzymes for fatty acid utilization, 5 out of 17 enzymes for BCAA utilization, 20 out of 47 enzymes for lactate utilization, 4 out of 5 enzymes for ketone body utilization, and 60/62 out of 127 enzymes for ATP production capacity under fasted/postprandial conditions as significantly associated with their respective metabolic functions (Fig. 5E). This plethora of significantly associated proteins indicates that metabolic capacities and cardiac dysfunction‐associated metabolic reprogramming is characterized by concerted regulation at the systems level rather than single proteins. Fig. 5F shows pyruvate kinase as a marker for the uptake capacity of glucose and Slc25Aa5 (adenine nucleotide translocator 2) as a marker for ATP production capacity. Both are not only functional markers but also show significant differences between the groups making them functional as well as disease‐specific markers. All pathway‐specific proteins with significant associations with metabolic capacities are given in Table S1.

### Fibrosis and inflammation

**Fig. 7:**
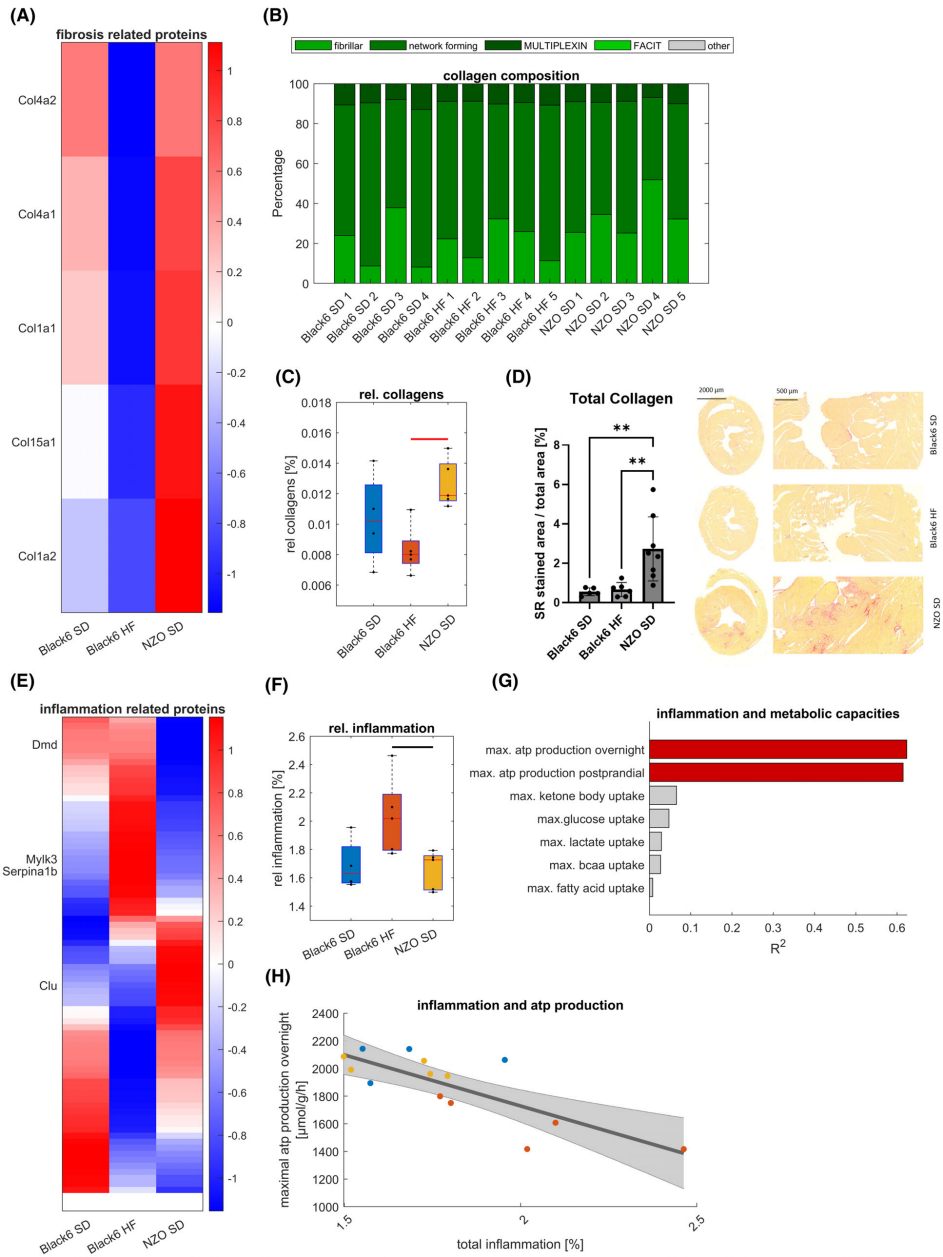
Fibrosis and inflammation. Sample sizes: Black6 SD (*n* = 4), Black6 HF (*n* = 5), and NZO SD (*n* = 5) for all panels except (D), where Black6 SD (*n* = 5), Black6 HF (*n* = 6), and NZO SD (*n* = 8). (A) *Z*‐score normalized mean protein intensities of collagen show an upregulation in the NZO SD mice and a downregulation in the Black6 HF mice. (B) Sample‐wise collagen composition. (C) Group‐wise comparison of collagen abundance derived from proteomic data (*P* = 0.0025). (D) Group‐wise comparison of total collagen content determined by Sirius Red (SR) staining. Bars represent mean ± SD, and individual points correspond to biological replicates. Data were tested for normality using the Shapiro–Wilk or Kolmogorov–Smirnov test. Statistical significance was determined by one‐way ANOVA followed by Tukey's multiple comparison test; **P < 0.01. Representative histological images from each group are shown on the right. The scale bar of the whole heart cross sections represents 2000 μm and of the heart tile scans 500 μm. (E) *Z*‐score normalized mean protein intensities of inflammation‐associated proteins show a heterogeneous distribution across the three groups. (F) Group‐wise assessment of total relative counts for inflammatory proteins shows a significant increase in the Black6 HF group compared to the NZO SD group (*P* = 0.027). Box plots in (C) and (F) indicate the median and interquartile range (25th–75th percentiles), with whiskers representing data variability and individual data points shown as black dots. Group values were checked for normality by a one‐sample Kolmogorov–Smirnov test. Statistical group differences were calculated by unpaired two‐sided Student's *t*‐test for normally distributed group values; otherwise, the Wilcoxon signed‐ranked test was used. Colored horizontal lines indicate statistically significant differences between groups (black; *P* < 0.05; red, *P* < 0.01). (G) Coefficient of determination (*R* ^2^) for the association between metabolic capacities and inflammation, calculated using linear regression analysis. Red bars indicate statistically significant associations (*P* < 0.05), and gray bars indicate nonsignificant associations (*P* ≥ 0.05). Each regression was performed separately for the indicated metabolic parameters using individual sample values. (H) Correlation between total intensity of inflammation‐associated proteins and maximal ATP production capacity under an overnight fasted condition. The black line indicates the linear regression fit, with the shaded gray area denoting the 95% confidence interval. Data points represent individual samples, color‐coded by group: Black6 SD (blue), Black6 HF (red), and NZO SD (yellow).

Structural changes and fibrosis are key components of impaired cardiac function [21]. Comparing protein abundances of collagens, Fig. 7A shows an upregulation of collagens in the NZO SD samples compared to a downregulation in the Black6 HF samples. Looking at the types of collagen shows a reduction in fibrillar collagen in the Black6 HF sample and a decrease in networking collagens in the NZO SD sample (Fig. 7B). Overall, there is a significant difference in the relative fraction of collagen between NZO SD and Black6 HF samples based on proteomics (Fig. 7C) and between NZO SD and Black6 HF as well as Black6 SD based on picrosirius red staining (Fig. 7D). Whereas the inhomogeneous distribution of inflammation throughout the heart might account for the discrepancy between proteomic and histological assessment of inflammation, our data show that the hearts of Black6 HF animals remained structurally healthy, while mild fibrosis developed in the NZO SD mice.

Finally, we investigated whether inflammation plays a role in both cardiac dysfunction models. Performing z‐normalized unsupervised clustering of proteins associated with inflammation by GO terms, we identified multiple differentially regulated clusters (Fig. 7E) with an overall significant upregulation of the total share of inflammatory proteins in Black6 HF compared to NZO SD mice (Fig. 7F). As it was shown previously that inflammation and metabolic alterations are linked in heart failure [22, 23], we performed linear regression analysis between the total share of inflammatory proteins and metabolic capacities including ATP production capacity. Fig. 7G,H shows that there is a highly significant negative association between inflammation and overall ATP production capacity under overnight fasted and postprandial conditions, but that there is no association with the utilization capacities of the energy‐delivering substrates. This indicates that inflammation directly acts on the ATP production machinery of the citric acid cycle and the respiratory chain.

### Association of metabolic capacities with cardiac function

**Fig. 8:**
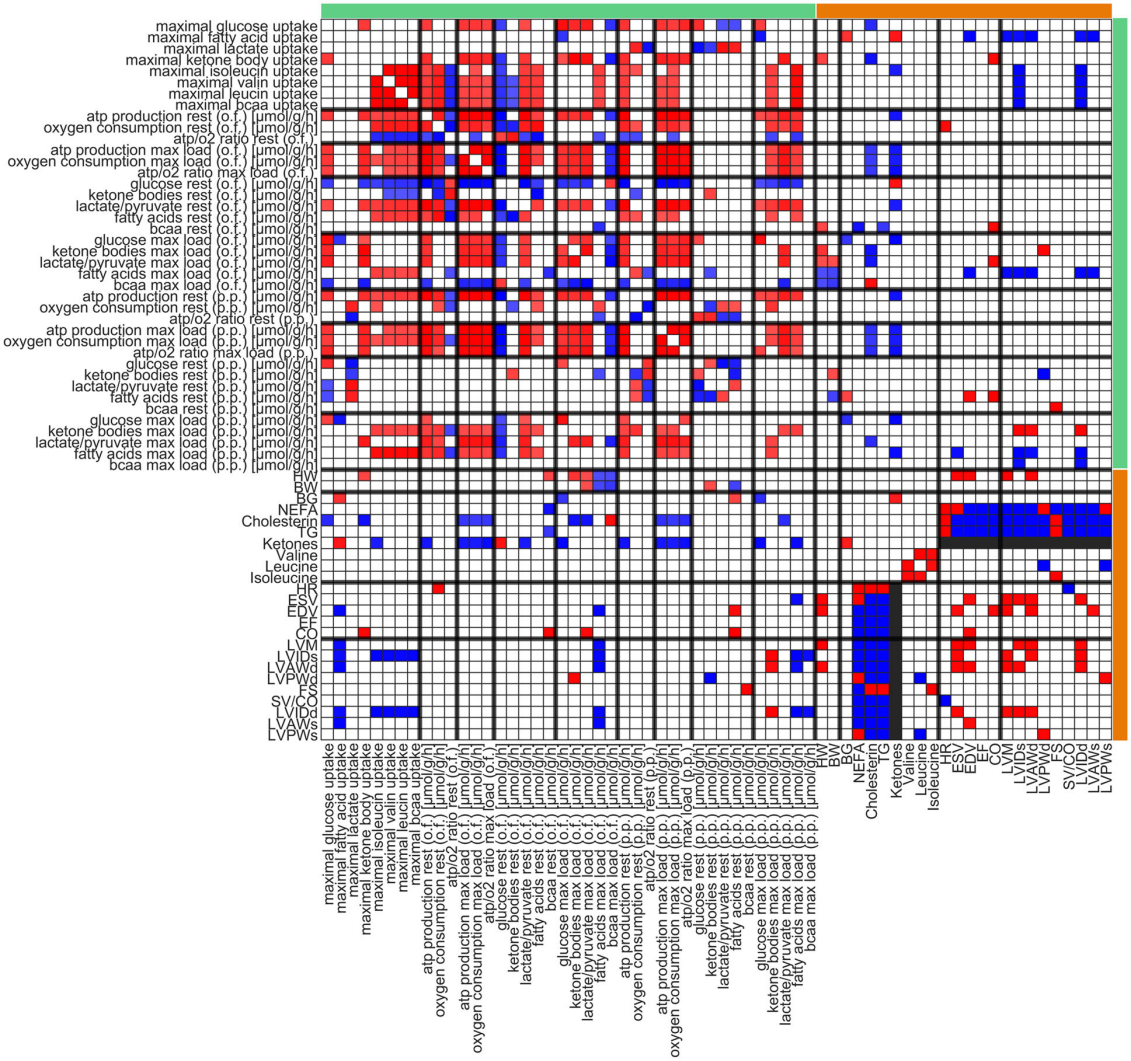
Significant correlations between metabolic capacities, plasma metabolites, and cardiac parameters in black 6 mice. Cardiac alterations in Black6 HF mice were analyzed in comparison to Black6 SD mice. Significant correlations between metabolic capacities (glucose, fatty acids, lactate, ketone bodies, isoleucine, valine, leucine, total bcaa, branched‐chain amino acids), ATP production (p.p., postprandial and o.f., overnight fasted, at rest and maximal energy demand), nutrient utilization, body weight (BW), and heart weight (HW), plasma nutrient composition (BG, blood glucose; NEFA, nonesterified fatty acids; TG, triglycerides; ketones, valine, leucine, isoleucine), and cardiac function and structure (HR, heart rate; ESV, end‐systolic volume; EDV, end‐diastolic volume; EF, ejection fraction; CO, cardiac output; LVM, left ventricular mass; LVIDs/d, LV inner diameter systole/diastole; LVAWs/d, LV anterior wall systole/diastole; LVPWs/d, LV posterior wall systole/diastole; FS, fractional shortening; SV(stroke volume)/CO) are shown. Blue indicates a significant negative correlation (*P* < 0.05), and red indicates a significant positive correlation (*P* < 0.05), determined by pairwise Pearson correlation analysis. Metabolic properties derived from proteomic‐based computational modeling are indicated by a green bar, and experimentally determined parameters by an orange bar.

**Fig. 9:**
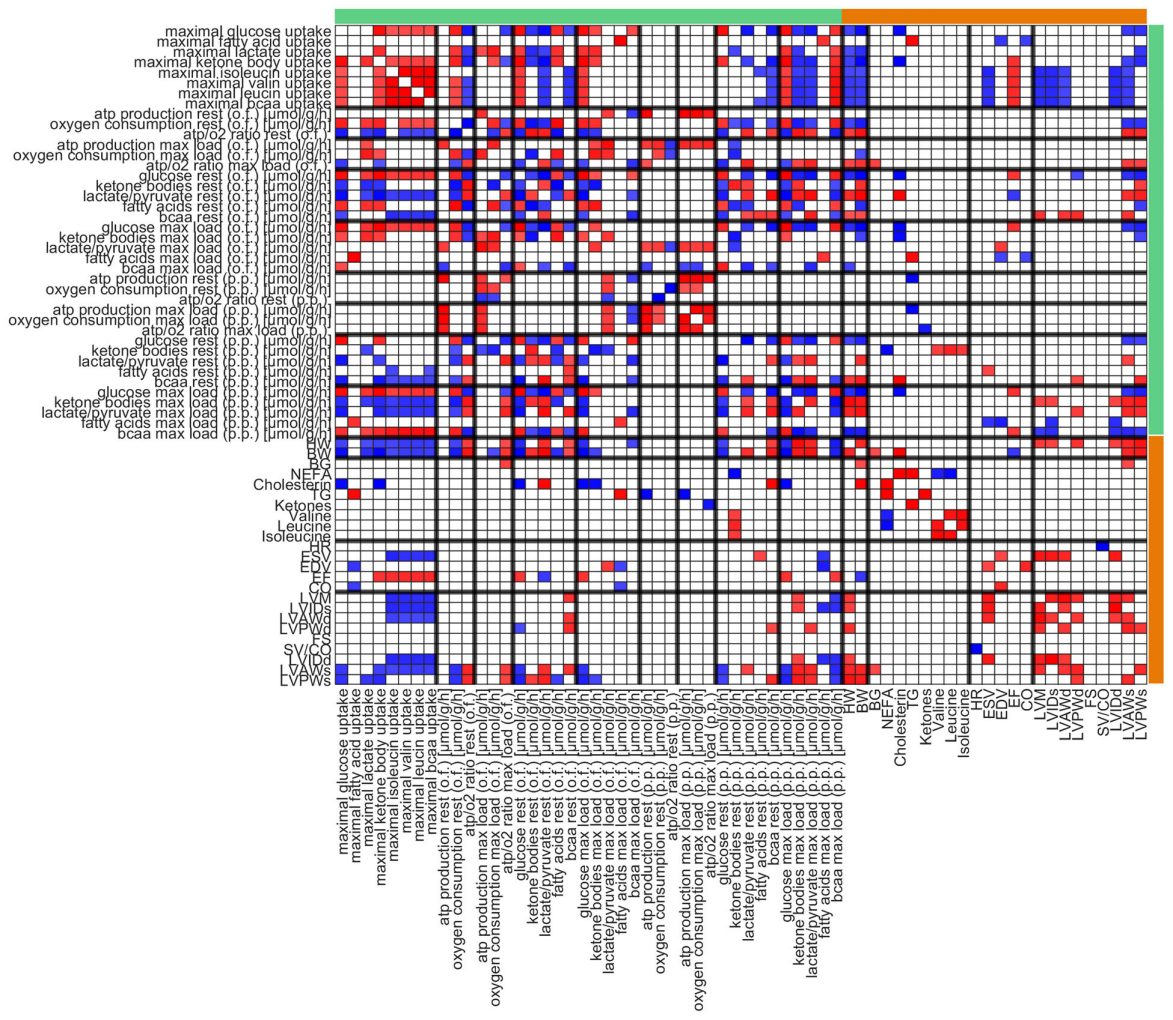
Significant correlations between metabolic capacities, plasma metabolites, and cardiac parameters in NZO mice. Cardiac alterations in NZO SD mice were analyzed in comparison to Black6 SD mice. Significant correlations were determined as described for Fig. 8, including metabolic capacities, ATP production (postprandial and overnight fasted, at rest and maximal energy demand), nutrient utilization, plasma metabolites, and cardiac function and structure parameters. Blue indicates significant negative correlations (*P* < 0.05), and red indicates significant positive correlations (*P* < 0.05). Green and orange bars denote model‐derived and experimentally measured parameters, respectively.

To reveal the underlying metabolic drivers of cardiac dysfunction in NZO SD and Black6 HF mice, we compared cardiac functional and structural parameters with the associated alterations in the metabolomic environment, proteomics, and metabolic capacities. While we found significant changes on all levels, it is informative to understand how these changes interact and are mutually dependent on each other. Figs 8 and 9 summarize the interdependencies of changes on the metabolic, nutritional, functional, and structural levels by looking at correlations between plasma metabolites, heart weight, body weight, and metabolic capacities, as well as structural adaptions in Black6 HF animals (Fig. 8) and NZO SD mice (Fig. 9). For example, in Black6 HF mice, the strong correlation between fatty acid utilization capacity and cardiac structural parameters suggests that impaired fatty acid handling may be a critical factor in the development of HFpEF. In contrast, the NZO SD model shows a predominant influence of body and heart weight on cardiac function, indicating that the metabolic adaptations in these mice are more closely related to obesity, eccentric cardiac hypertrophy, and fibrosis, typical of HFrEF. These insights are crucial for understanding the distinct metabolic pathways involved in each cardiac dysfunction phenotype and could guide targeted therapeutic strategies.

In Black6 HF mice, fatty acids dominate as an energy source, aligning with the reduced capacity for glucose uptake, likely due to the high dietary availability of fatty acids and the absence of exogenous carbohydrates. The negative correlation indicates that glucose is replaced by other nutrients under resting conditions. A similar pattern is observed in NZO SD mice, where fatty acids and lactate compete with glucose. However, a key difference is the decoupling of glucose utilization at rest from overall substrate utilization capacity. Still, these capacities do not represent physiological resting conditions as they demand hyper‐physiological substrate concentration while largely omitting competing substrates. For example, despite being available, lactate is not a primary energy source under resting conditions; instead, fatty acids and glucose are preferentially used. Lactate only becomes relevant at high‐energy demands in Black6 SD and NZO SD mice. In Black6 HF mice, mitochondrial impairment and reduced oxidative phosphorylation capacity mean that fatty acids and glucose sufficiently utilize the respiratory chain, preventing lactate utilization (see Fig. 5).

In addition to the many interdependencies within the block of cardiac metabolic functions (comprising metabolic capacities for the utilization of the different energy‐delivering substrates, ATP production rates, oxygen consumption, and nutrient utilization), there is a very strong correlation between plasma fatty acids and TG, and structural as well as functional alterations (heart rate, ESV, EDV, EF, CO) in the hearts of Black6 HF animals (Fig. 7A). Consistently, the capacity for fatty acid utilization is the main indicator for changes in cardiac structure (significant negative association with LVM, LVIDs, LVAWd, LVIDd, LVAWs), which is also mirrored by the fatty acid utilization rate at maximal load under overnight fasted conditions (i.e., fatty acid utilization has to be maximal to fulfill energetic demand), underpinning elevated fatty acids and alterations in fatty acid handling as potential underlying triggers of cardiac dysfunction.

However, alterations in fatty acids and fatty acid handling are not relevant in the NZO SD mice, where body weight and heart weight are the main indicators of functional cardiometabolic alterations (Fig. 7B). Increased body and heart weight go along with a decreased capacity to utilize any energy‐delivering substrate (except for fatty acids), a decreased ATP production capacity, shifts in substrate preferences, and structural alterations. Of the functional parameters, only EF is negatively associated with increased body weight. Noteworthy, there are almost no correlations between plasma nutrients and structural, functional, or metabolic properties of the failing heart. Across both animal models, only a few structural changes are associated with functional changes.

## Discussion

Recent advances in cardiac metabolism research provide valuable insights into the functional, structural, and metabolic underpinnings of HFpEF versus HFrEF [1, 2, 3]. Understanding these differences is crucial for developing targeted interventions and therapeutic strategies.

In our study, C57BL/6J mice were subjected to a high‐fat, no‐carbohydrate diet (Black6 HF) to specifically induce metabolic inflexibility of the heart through diet, while NZO mice on a standard diet (NZO SD) served to mimic the development of spontaneous obesity and cardiac dysfunction with reduced EF [14, 16]. The high‐fat diet (HF) used in this study differs from a typical Western diet, particularly in its composition of fats and absence of carbohydrates. It is designed to mimic extreme metabolic stress by providing 30.5% of energy from fat without any carbohydrate sources, whereas a Western diet typically includes both high‐fat and high‐carbohydrate content, leading to different metabolic responses [24, 25]. While the lack of carbohydrates and the increase in dietary fatty acids may resemble certain aspects of a ketogenic diet, our data do not support a ketogenic state in these mice. Specifically, we observed no significant increases in circulating ketone bodies or decreases in circulating glucose levels. This indicates that the body—particularly the liver and kidneys—can maintain systemic nutrient availability through gluconeogenesis, effectively buffering the metabolic impact of the dietary changes without relying on ketogenesis.

Unexpectedly, the differences in diet composition likely contribute to the distinct metabolic and functional outcomes observed in Black6 HF through altered fatty acid metabolism and reduced glucose utilization. The lack of significant body mass increase in Black6 HF mice, despite the HF, can be attributed to the absence of carbohydrates in the diet. High‐fat, carbohydrate‐free diets promote a metabolic shift towards fat oxidation, which prevents excessive fat storage and weight gain. Contrarily, including carbohydrates in the HF led to a significant body mass increase, yet still resulted in similar cardiac dysfunction characteristics (data not shown). These findings underscore the critical role of diet composition in modulating both metabolic outcomes and the development of cardiac impairment, highlighting the importance of dietary macronutrient balance in experimental models of cardiometabolic disease.

### Structural and functional implications: Diet and genetically induced cardiac dysfunction

Echocardiographic assessments showed significant cardiac functional impairments in both models, albeit with unique phenotypic peculiarities. The Black6 HF mice demonstrated decreased SV and EDV while maintaining a preserved EF, indicating a completely different phenotype compared to the genetically induced cardiac dysfunction in the NZO mice.

NZO SD mice exhibited a significant increase in ESV and an increase in LVIDd, alongside a decreased EF to around 40%, typical of the systolic dysfunction seen in HFrEF [7]. The changes included increased myocardial wall thickness and ventricular dilation and were also accompanied by significant cardiac weight gain and fibrosis. These structural differences are critical as both hypertrophy and fibrosis can further impair diastolic function, a common feature of heart failure [21]. Surprisingly, heart weight and left ventricular mass were decreased in Black6 HF, whereas they were increased in NZO SD compared to control mice, suggesting that changes in heart mass do not directly correlate with functional impairment. This suggests that factors other than cardiac mass, such as metabolic flexibility and mitochondrial function, play a critical role in maintaining cardiac function. The reversibility of these changes remains uncertain, but existing literature suggests that interventions targeting metabolic pathways, such as those enhancing mitochondrial function or reducing metabolic stress, may partially reverse structural changes [26].

### Metabolic pathways and cardiac dysfunction

Heart failure is characterized by significant alterations in energy substrate utilization where the failing heart undergoes a metabolic shift from fatty acid oxidation to increased reliance on glucose metabolism, both in HFrEF and HFpEF [2, 3]. The metabolic shifts seem to become more pronounced with increasing severity of heart failure, but also contradicting findings have been reported [27].

The capacity for substrate utilization at supraphysiological concentrations was reduced for glucose, lactate, ketone bodies, and branched‐chain amino acids in NZO mice, and for glucose and ketone bodies in Black6 HF mice. While these changes indicate metabolic impairment in both models, the overall cardiac metabolic capability under physiological conditions—assessed by ATP production capacity in the presence of all substrates and regulatory interactions—remained preserved in NZO SD mice but was significantly reduced in Black6 HF mice. In a healthy heart, the maximal ATP production capacity exceeds resting energy demand by 3‐4‐fold [15]. This reserve capacity was maintained in NZO mice, even though their maximal metabolic capacities for most individual substrates were reduced. While individual substrate reserve capacities were limited in NZO SD mice, the combined utilization of substrates was sufficient to sustain the heart's respiratory and energy demands. In contrast, the severely reduced metabolic capacity of Black6 HF mice indicates a compromised ability to meet ATP production demands, hinting to mitochondrial dysfunction.

Cardiac efficiency, defined as the ratio of cardiac work to oxygen consumption (ATP/O_2_ ratio), suggests reduced efficiency in the Black6 HF model, likely due to impaired mitochondrial function and a compensatory switch to oxygen‐independent glycolytic ATP production. While uncoupling proteins (UCPs), particularly UCP2 and UCP3, are known to modulate mitochondrial efficiency by dissipating the proton gradient, thereby reducing ATP production efficiency [28], neither was found in the proteomic data. Our data show a clear reduction in the capacity to utilize glucose and a shift in substrate preference towards fatty acid utilization. The upregulation of fatty acid uptake and utilization proteins in the Black6 HF mice likely reflects an adaptative mechanism in response to increased fatty acid availability from the HF. This is also demonstrated by the mean increase in fatty acid utilization capacity in the Black6 HF group. However, the adaption is heterogeneous, leading to a nonsignificant overall increase.

Especially at high‐energy demand under postprandial conditions, we observed a clear reduction in glucose utilization in the NZO SD cohort indicating a loss of metabolic flexibility despite maintaining overall ATP production capacity.

Our study also shows that the diet‐induced cardiac dysfunction was associated with minimal changes in basal fatty acid and glucose utilization, whereas the genetically induced phenotype (in NZO SD mice) exhibited significant alterations in the substrate preference under resting conditions (the relative contribution of glucose to overall ATP production was reduced by ~50% under overnight fasted conditions).

### Mitochondrial dysfunction

One of the main underlying reasons for these metabolic shifts especially at high‐energy demand is mitochondrial dysfunction, which classically is regarded to be more severe in HFrEF while HFpEF is considered to have milder mitochondrial impairments [29]. The mitochondria dysfunction‐associated hearts are characterized by decreased oxidative phosphorylation efficiency, reduced ATP output, and increased oxidative stress [30].

In our study, the Black6 HF cohort showed a significant reduction in overall ATP production capacity indicating mitochondrial dysfunction, as oxidative phosphorylation is significantly reduced. On the substrate level, this is also very clearly shown by the inability to utilize lactate as an energy‐delivering substrate, as oxidative phosphorylation capacity is already saturated with fatty acids (NEFAs) and glucose. Indeed, it has been shown that lactate is a major energy source under stress conditions [31] and increased lactate levels are an indicator of heart failure in patients [32, 33], and therefore, the inability to use lactate might be an important indicator of cardiac dysfunction. Reduced ketone body uptake capacity observed in the Black6 HF and NZO SD mice is indeed unexpected, given the reported reliance on ketones as an alternative energy source in heart failure [27]. However, the notion that ketone bodies represent an alternative fuel during heart failure makes only sense under conditions of increased ketone body availability, which is not true for our animal models. Overall, ketone bodies are only contributing insignificantly to overall ATP production making even a relative increase noneffective for ATP production.

The reduction in ATP production capacity in the Black6 HF mice, but not in the NZO SD mice, likely reflects the severe mitochondrial dysfunction induced by the HF. In contrast, the NZO SD mice maintain ATP production capacity despite significant shifts in substrate preference, possibly due to compensatory upregulation of glucose metabolism pathways. This suggests that while both models exhibit metabolic inflexibility, the underlying mechanisms differ, with mitochondrial efficiency being more compromised in the diet‐induced cardiac dysfunction model [34].

Importantly, the metabolic derangement is also mirrored in the plasma nutrient composition, where significant alterations especially in fatty acids, the main energy‐delivering substrate for the heart are altered only in the diet‐induced model but not in the NZO SD mice.

The reduction in CO/BW by ~50% in Black6 HF mice and by ~67% in NZO SD mice compared to Black6 SD controls together with the metabolic derangements suggests that cardiac function may be insufficient to meet systemic demand. However, no hypoxic markers such as hypoxia‐inducible factor 1α or Bcl‐2 19‐kDa interacting protein 3 were detected in the samples using the proteomic data. While in Black6 HF mice, the ~50% reduction in CO with unchanged body weight together with the significant impairment in cardiac ATP production capacity might hint at a profound loss of cardiac reserve capacity, in NZO SD mice, the greater reduction in CO/BW reflects both impaired cardiac function and increased body weight. For each metabolic pathway, we identified multiple marker proteins, which are directly linked to cardiac dysfunction. In ATP production, the identified proteins include key dehydrogenases of the citric acid cycle, such as pyruvate dehydrogenase, isocitrate dehydrogenase, α‐ketoglutarate dehydrogenase, and succinate dehydrogenase, along with multiple subunits of respiratory chain complexes I, II, and III, ATP synthase, and the adenine nucleotide translocator. A reduction of the activity of citric acid cycle dehydrogenases and respiratory chain components has been reported in various heart failure models [35, 36, 37] and in humans [38, 39, 40]. Additionally, key enzymes of fatty acid metabolism directly linked to cardiac function, including acyl‐CoA dehydrogenase [41], acyl‐CoA synthetase [42], and carnitine palmitoyl transferase [43], were identified. Regarding glucose metabolism, we identified essential enzymes of the glycolytic pathway that have been associated with heart failure, such as phosphofructokinase [44], enolase [45], as well as GAPDH and phosphoglycerate kinase [46]. Moreover, lactate dehydrogenase has been suggested as a potential prognostic marker [47].

### Implications for therapeutic targeting: Metabolic enzyme modulation

The proteomic analysis underscores this point, showing a distinct pattern of mitochondrial protein downregulation in Black6 HF but not in NZO SD mice compared to those of the control group. Importantly, the underlying alterations in protein abundances are not limited to single enzymes but reflect coordinated shifts in the underlying metabolic network as the expression levels of around half of the enzymes involved in ATP production (i.e., citric acid cycle, respiratory chain, oxidative phosphorylation) are directly associated with the energetic capacity.

Given the distinct metabolic profiles of the examined cardiac dysfunction phenotypes, targeting specific metabolic enzymes and pathways offers a promising therapeutic strategy. For instance, enhancing fatty acid oxidation or optimizing glucose utilization through pharmacological agents could potentially alleviate the energy deficit. Drugs like perhexiline, which modulates lipid metabolism, have shown some promise in improving cardiac function by normalizing metabolic substrate utilization [48, 49]. Importantly, they are supposed to work by stimulating glucose metabolism through inhibiting fatty acid oxidation, thereby shifting substrate utilization towards healthy conditions, which is exactly coinciding with the metabolic shifts observed in our diet‐induced model. However, for the NZO model, stimulation of oxidative phosphorylation should be more beneficial. Unloading the heart from metabolic stress through interventions such as bariatric surgery, fasting, or pharmacological treatments like SGLT2 inhibitors, GLP‐1 agonists, or metformin has been proposed as a strategy to mitigate heart failure [50, 51, 52]. These interventions work by improving insulin sensitivity, promoting weight loss, and reducing metabolic stress, thereby potentially reversing some of the metabolic and structural changes observed in heart failure. In our study, such interventions could be particularly relevant for the Black6 HF model, where reducing fatty acid load and improving glucose utilization might restore metabolic flexibility and improve cardiac function.

### Limitations

A key limitation of this study is the incomplete characterization of the cardiac phenotype in Black6 HF. We have not yet evaluated all key clinical parameters of diastolic function or exercise intolerance. This would have provided additional insights into whether this specific dietary intervention can induce HFpEF beyond the observed maintained EF and impaired cardiac output. Furthermore, epicardial fat, known to contribute to cardiac inflammation and dysfunction, was not measured in this study. However, given its established role in heart failure, particularly in obesity‐related conditions, future studies could assess epicardial fat to better understand its contribution to the metabolic and structural changes observed in our models [53]. Differences in the epicardial fat deposition could potentially explain some of the observed differences between the diet‐induced and genetically induced cardiac dysfunction models. Furthermore, the inclusion of lung weight in future studies could provide further valuable insights into pulmonary congestion and the severity of cardiac impairment in the context of metabolic changes.

While our computational modeling approach using CARDIOKIN1 provides quantitative predictions of cardiac metabolic capacities based on proteomic data, we acknowledge that it does not directly assess metabolic fluxes. Although our analysis infers potential changes in substrate utilization and ATP production capacity, only direct flux measurements—such as those obtained through stable isotope tracing or radiolabeled substrates—can conclusively determine how these capacity changes translate into dynamic pathway activity [54]. In addition, mitochondrial activity can be assessed by measuring oxygen consumption. This represents a technically accessible and functionally informative proxy for ATP‐generating capacity and serves as a valuable control to validate the functional relevance of model‐inferred changes in energy metabolism. This combined approach could not only validate model‐based predictions but also help identify critical control points within metabolic pathways in cardiac dysfunction.

### Conclusion

Distinct metabolic changes in different forms of cardiac dysfunction, as elucidated through advanced proteomic and computational analyses, highlight the importance of a nuanced understanding of cardiac energy metabolism in cardiac impairment. In this study, we aimed to investigate how heart function and metabolism adapt to a high‐fat, noncarbohydrate diet compared to spontaneously occurring obesity driven by genetic predisposition. By successfully integrating heart proteomic data into the CARDIOKIN1 model, we enabled a unique comparison of dietary and genetic influences on cardiac adaptations. While NZO mice developed cardiac dysfunction with reduced EF, an unexpected and intriguing finding was the response in Black6 HF mice, which exhibited mitochondrial dysfunction and reduced ATP production capacity as key drivers of cardiac impairment despite maintaining EF.

Our findings highlight that shifts in metabolic capacity in response to substrate availability and genetic predisposition play a critical role in cardiac dysfunction phenotyping. Metabolic inflexibility may contribute to cardiac dysfunction by impairing energy production and adaptation to physiological stress, underscoring the importance of substrate supply and utilization in shaping the diverse phenotypes of cardiac impairment. Distinct metabolic and cardiac signatures observed in models of diet‐ and genetically induced cardiac dysfunction highlight the necessity of tailored therapeutic strategies that address specific metabolic dysfunctions. Delineating these differences not only increases our understanding of disease mechanisms but also opens up new avenues for targeted metabolic therapies either through pharmacological or nutritional interventions. Integrating multi‐omics data with computational models will help to identify key metabolic nodes for future nutritional or pharmaceutical interventions. Implementing this approach in model systems could result in clinical trials targeting pre‐identified metabolic pathways, including those regulating fatty acid and glucose metabolism, ultimately facilitating the translation of these findings into effective treatment strategies for heart failure patients.

## Materials and methods

### Animals

We used the ARRIVE reporting guidelines throughout the manuscript [55]. All animal procedures were performed in accordance with the German Law on the Protection of Animals. The study was reviewed and approved by the local authorities (Landesamt für Gesundheit und Soziales, Berlin (LAGESO) and Landesamt für Arbeitsschutz, Verbraucherschutz und Gesundheit Brandenburg, Potsdam (LAVG), Germany; approval number: 2347–5‐2017 LAVG/G0239/16 LAGESO). All mice were randomly rearranged and assigned to open cage housing of 4–5 animals in a controlled environment (20 ± 2 °C, 12/12 h light/dark cycle) with *ad libitum* access to food and water. From the age of five weeks, male black 6 mice (C57BL/6JRj, Janvier Labs, Saint Berthevin Cedex, France) received either a standard diet in the control group (Black6 SD) (V1534‐300 Ssniff, Soest, Germany, for 17 weeks) or were fed with a high‐fat no‐carbohydrate diet (Black6 HF) (#C105789, Altromin, Lage, Germany, containing 32.1% (wt/wt) protein and 30.6% (wt/wt) fat, for 13 weeks). To metabolically compare Black6 SD (control group) and Black6 HF to a HFrEF‐like phenotype, we included the NZO mouse (NZO/HIBomDife, German Institute of Human Nutrition Potsdam‐Rehbruecke, Germany), known to develop obesity and cardiac impairment already on SD, as previously described by us [14]. Male NZO mice were housed under the same conditions as black 6 and received the same SD as Black6 SD for 17 weeks. The details of the diets are listed in Table 1.

**Table 1:** Nutrition facts of the standard diet (SD) and carbohydrate‐free high‐fat diet (HF).

Diet		SD (V1534‐300 Ssniff, Soest, Germany)		HF (#C105789, Altromin, Lage, Germany)	
		[%]	[mg/kg]	[%]	[mg/kg]
Fat	Crude fat	3.3	33 000	30.5	305 580
	SFA	0.56	5600	2.6	26 255
	MUFA	0.63	6300	8.1	81 715
	PUFA	1.99	19 900	0.088	885,00
Protein	Crude protein	19	190 000	32	320 790
	Leucine	1.39	13 900	1.9	19 018
	Valine	0.92	9200	0.7	7401
	Isoleucine	0.79	7900	0.92	9214
Carbohydrates	Crude fiber	4.9	490	20	201 891
	Starch	35.2	352 000	‐	‐
	Sugar	5.3 *54.2	53 000 *540 000	‐	‐
Sodium		0.24	2400	0.27	2761
Metabolized energy (kcal/kg)	3230 (w/67% energy from carbohydrates)			4081 (w/60% energy from 30.5% fat)	

* *N*‐free extractives.

Abbreviations: MUFA, monounsaturated fatty acids; PUFA, polyunsaturated fatty acids; SFA, saturated fatty acids.

To control all animals regarding the development of a severely obese and diabetic phenotype (exclusion criteria), body weight and blood glucose were measured weekly. At the age of 18 or 22 weeks, echocardiographic analysis was performed and changes in cardiac function (e.g., cardiac output) due to the diets were analyzed. Before echocardiography was performed, the animals were always allowed an acclimatization period of at least one week. Blood glucose was measured using blood obtained from a tail incision with a Contour blood glucose meter and Contour NEXT Sensors (Ascensia Diabetes Care Deutschland GmbH, Leverkusen, Germany). At the end of the dietary intervention, mice (18–22 weeks of age) were sacrificed in the morning, without an overnight fast, by acute isoflurane exposure, and blood and tissue samples were collected for proteomics and molecular analysis.

### Data acquisition methods

Data acquisition was randomized and blinded. Final measure units received only sample numbers without assignment. Evaluation and assignment were performed independently.

#### Echocardiography

To assess cardiac morphology and function, echocardiography was carried out at the ages of 18 and 22 weeks. Echocardiography of the left ventricle (LV) was performed using a Vevo 3100 high‐resolution Imaging System coupled to an MX400 ultra‐high‐frequency linear array transducer (18–38 MHz; center transmit: 30 MHz; axial resolution: 50 μm) (both FUJIFILM VisualSonics, Toronto, Ontario, Canada), as described previously [56, 57]. In brief, mice were exposed to 3% isoflurane (Baxter International, Deerfield, IL) and fixed in a dorsal position on a heating pad maintaining physiological body temperatures during the echocardiography recording. For maintenance of anesthesia, isoflurane levels were reduced to 1–2%. M‐Mode images of the LV in parasternal long axis view were used to characterize diastolic and systolic wall thicknesses and to calculate left ventricular mass (LVM) and LV function parameters, for example, ejection fraction (EF) using the dedicated software package VevoLab (VisualSonics).

#### Blood plasma parameter

In collected blood samples at the study end, plasma triglycerides, nonesterified fatty acids (NEFAs), and ketone levels were measured by a colorimetric assay according to the manufacturer's instructions using the following analytic kits and controls: (for ketones) RANBUT D‐3‐Hydroxybutyrate Randox‐Reagent (RB1007, Labor Technik Lehmann, Berlin, Germany); (for NEFAs) NEFA‐HR (2) R1 Set R1a, NEFA‐HR (2) R2 Set R2a, NEFA STANDARD (434‐91795, 436‐91995, 270‐77000 wako Fujifilm, Neuss, Germany); and (for triglycerides) Triglyceride Reagent (A11A01640, Axonlab, Reichenbach, Germany), with ABX Pentra 400 benchtop analyzer (Horiba, Kyoto, Japan). Amino acid analysis was performed as described by Henning *et al*. [58]. In brief, 10 μL of plasma were mixed with 40 μL internal standard working solution, consisting of 11 deuterated amino acids, and vortexed for 30 s. For protein precipitation, samples were stored at −20 °C for 10 min, followed by centrifugation at 30.000 × ***g***, 4 °C, 10 min. After the transfer of 40 μL supernatant into a vial with a 100 μL glass insert, 2 μL was injected into the LC system, and separation of amino acids was performed using an ACQUITY UPLC BEH Amide column (2.1 mm × 100 mm, 1.7 μm) equipped with a VanGuard BEH Amid precolumn (2.1 mm × 5 mm, 1.7 μm). Mobile phase A consisted of acetonitrile/Milli‐Q water (9 : 1, v/v) containing 5 mm ammonium formate and 0.3% formic acid (v/v). Mobile phase B consisted of Milli‐Q water containing 25 mm ammonium formate (pH 6). Gradient elution started at 98% solvent A and was held for 4 min. The proportion of solvent B was increased to 25% within 0.5 min. To start, 25% B was maintained between 4.5 and 6.5 min, and solvent B was increased to 40% within 0.1 min. The 40% B solvent was kept constant between 6.6 and 8.0 min. Finally, solvent B was reduced to 2% within 0.1 min and the column was re‐equilibrated with initial conditions for another 1.9 min, leading to a total runtime of 10 min. The flow rate was constantly set at 0.4 mL·min^−1^. Samples were kept in the autosampler at 6 °C, and the column temperature was set at 35 °C. Mass spectrometer Xevo TQ‐MS (Waters Corporation, Eschborn, Germany) was operated in ESI‐positive mode. The desolvation temperature was set at 600°C, and the desolvation gas flow was set at 600 L·h^−1^. Source capillary voltage was set at 0.5 kV, cone gas flow was set at 150 L·h^−1^, and nebulizer gas pressure was set at 7.0 bar.

#### Picrosirius red staining

For picrosirius red staining, hearts were fixed in 4% formalin and embedded in paraffin. Paraffin slices (cross section, 2 μm) were deparaffinized via a descending ethanol series and subsequently stained with picrosirius red solution for 1 h. After staining for 1 h, slides were washed twice with 0.5% acetic acid for a few seconds before dehydration in an ascending ethanol series. For imaging or representative images, the Axio Scan 7 (Zeiss) with a 20× objective was used.

### Proteomics sample preparation

Tissue samples were homogenized in 500 μL lysis buffer (8 M urea in 20 mm HEPES) using a mechanical shredder, followed by centrifugation to remove the foam. The homogenate was transferred to a new tube and sonicated on ice at 20% amplitude, three 10‐s bursts until no longer viscous. Benzonase® HC nuclease (Millipore, 71‐206, 0.5 units per 1 μg of protein) was added, and the samples were incubated at 37°C for 30 min followed by centrifugation at 20000 ***g*** for 15 min at room temperature. Afterward, DTT was added to a final concentration of 5 mm, and the samples were incubated in a thermoshaker at 55 °C, 700 rpm for 30 min. After briefly cooling the samples on ice to room temperature, iodoacetamide was added to a final concentration of 10 mm for alkylation. Therefore, the samples were incubated in the dark at 700 rpm for 15 min. Subsequently, the protein concentration was measured using the Pierce 660 nm protein assay (Art. 22 662), and the samples were diluted to equal concentrations (e.g., 1 mg·mL^−1^). Afterward, the samples were diluted with 20 mm HEPES to a final urea concentration of 4 M. Lys‐C (1 μg·μL^−1^ stock solution in water) was added at a 1 : 100 (w : w) ratio, and the samples were incubated at room temperature for 4 h with shaking. The samples were then further diluted with 20 mm HEPES to reduce the urea concentration to 2 M, after which trypsin (1 μg·μL^−1^ stock solution in 20 mm HEPES) was added at a 1 : 100 (w : w) ratio. Digestion was carried out overnight at room temperature with shaking. The following day, the samples were acidified to a final concentration of 1% trifluoroacetic acid (TFA) and adjusted to a pH < 3. After a 15‐min precipitation on ice, the samples were centrifuged at 1780 ***g***, 15 min at room temperature, and the supernatant was transferred to a new tube for peptide purification. Peptides were purified using SampliQ C18 100 mg columns. The columns were prewashed with 1 mL of 100% acetonitrile (ACN), followed by 3 mL of Wash Buffer (2% ACN, 0.1% TFA). The acidified and cleared digest (up to 1 mg peptide material) was loaded onto the column, followed by a 2 mL wash with Wash Buffer. Peptides were eluted with two applications of 750 μL Elution Buffer (60% ACN, 0.1% TFA). Eluted samples were dried in a SpeedVac concentrator for approximately 5 h.

### Shotgun proteome profiling and data analysis

Liquid chromatography with tandem mass spectrometry (LC–MS/MS) was carried out by nanoflow reverse‐phase LC (Dionex Ultimate 3000, Thermo Fisher Scientific, Waltham, MA, USA) coupled online to a Q Exactive Plus Orbitrap mass spectrometer (Thermo Fisher Scientific). Peptide mixtures were fractionated by an Ultimate 3000 RSLCnano (Thermo Fisher Scientific) with a two‐linear‐column system. Digests were concentrated onto a trapping guard column (PepMap C18, 5 mm x 300 μm × 5 μm, 100Ǻ, Thermo Fisher Scientific). Then, samples were eluted from the analytical column a 75 μm i.d. × 250 mm nano‐LC column (Acclaim PepMap C18, 2 μm; 100 Å; Thermo Fisher Scientific). Separation was achieved by using a mobile phase from 0.1% formic acid (buffer A) and 80% acetonitrile with 0.1% formic acid (buffer B) and applying a linear gradient from 8 to 28% of buffer B for 60 min at a flow rate of 300 nL·min^−1^. Nanoelectrospray was generated by applying 2.1 kV. A cycle of one full Fourier transformation scan mass spectrum (350–1650 *m/z*, resolution of 70 000 at *m/z* 200, AGC target 3*1e6) was followed by 10 data‐dependent MS/MS scans (resolution of 17 500, AGC target 5e4) with a normalized collision energy of 27%. To avoid repeated sequencing of the same peptides, a dynamic exclusion window of 10 s was used. Only the peptide charge states *z* ≥ 2 were sequenced.

Raw MS data were processed with MaxQuant software (1.6.0.1) [59] with the Andromeda search engine, and spectra were matched to a *Mus musculus* database (17 040 reviewed entries, downloaded from uniprot.org in March 2020), a contaminant, and decoy database. A false discovery rate of 0.01 for proteins and peptides, a minimum peptide length of seven amino acids, a mass tolerance of 10 ppm for precursor, and 20 ppm for fragment ions were required. A maximum of two missed cleavages was allowed for the tryptic digest. Cysteine carbamidomethylation was set as a fixed modification, while deamidation (NQ) and oxidation (M) were set as variable modifications.

### Assessment of metabolic capacities based on proteomic data

For the quantification of the metabolic changes caused by the abundance changes of metabolic enzymes, we used the kinetic model CARDIOKIN1. The model was introduced and described in detail previously [15] and has been applied in other studies for the analysis of cardiac metabolic adaptations (e.g., [18, 60, 61]). It comprises all pathways involved in the catabolism of the energy‐delivering substrates glucose, lactate, fatty acids (NEFAs), ketone bodies, and branched‐chain amino acids (BCAAs) as well as synthesis of endogenous energy stores (glycogen, triacylglycerol). The model takes into account the regulation of metabolic enzymes and transporters by substrate affinities, allosteric regulations as well as the short‐term regulation by the hormone insulin and catecholamines. Individual model instantiations were generated based on proteomic profiles as described in Berndt *et al*. [62].

Maximal metabolic capacities were defined by the largest obtainable magnitude of fluxes in response to changes in the concentration of distinct plasma metabolites while keeping all other plasma metabolites at a constant level. For a detailed description, see Berndt *et al*. [15].

Energetic capacities were assessed under two physiological conditions: a postprandial state with high glucose and low fatty acid concentrations, and a fasted state with low glucose and high fatty acid concentrations given in Table 2.

**Table 2:** Physiological plasma profiles used for evaluation of ATP production capacities.

Metabolites	Postabsorptive state (overnight fasted)	Postprandial state (after meal)
Glucose	5.8 mm	7.8 mm
Fatty acids	0.5 mm	0.1 mm
Lactate	0.8 mm	2.0 mm
Valine	0.2 mm	0.4 mm
Leucine	0.15 mm	0.4 mm
Isoleucine	0.06 mm	0.2 mm
β‐hydroxybutyrate	0.08 mm	0 mm
Acetoacetate	0.04 mm	0 mm
Catecholamines	0.75 nm	0.75 nm
Insulin	100 pm	600 pm

The metabolic response of the samples to an additional ATP demand was evaluated by computing the temporal changes of the metabolic state elicited by an increase in the ATP consumption rate above the resting value. The ATP consumption rate was modeled by a generic hyperbolic rate law $v_{\mathrm{ATP}}=\text{load}\cdot \frac{\mathrm{ATP}}{\mathrm{ATP}+K_{m}}$. The parameter $k_{\text{load}}$ was stepwise increased until the ATP production rate converged to its maximal value. For further details, see Berndt *et al*. [15].

### Statistical analysis

Statistical analysis, cluster analysis, and Pearson correlation analysis were performed using MATLAB Release 2021a with the bioinformatics toolbox (The MathWorks, Inc., Natick, MA, USA). Cluster analysis was performed using the clustergram function. The values of the calculated metabolic functions were transformed to mean ‘0’ and standard deviation ‘1’. Group values were checked for normality by a one‐sample Kolmogorov–Smirnov test. Statistical group differences were calculated by unpaired two‐sided Student's *t*‐test for normally distributed group values; otherwise, the Wilcoxon signed‐ranked test was used. Linear regression models were performed to assess the association of metabolic capacities with functional and proteomic parameters. A value of *P* < 0.05 was taken as statistically significant.

Values of physiological and echocardiographic parameters are shown as mean ± standard deviation. Statistical analyses were performed using repeated measures ANOVA with Tukey's or Kruskal–Wallis multiple comparisons test as appropriate. A difference with *P*‐value <0.05 was assumed as statistically significant. After corroborating the Gaussian distribution of the data by using the D'Agostino Pearson test, Kolmogorov–Smirnov, or Shapiro–Wilk normality test, the correlation of cardiovascular metrics was tested with the Pearson correlation coefficient (*r*). All analyses were performed using graphpad prism 9 (graphpad software, La Jolla, CA).

## Author contributions

Conceptualization: TG and NB; methodology: CO, TH, UK, and NB; formal analysis: CO, PB, TH, JG, HGH, and NB; investigation: CO, TH, DW, and JG; resources: UK, TG, and NB; data curation: CO, PB, IW, and NB; project administration: IW; funding acquisition: UK, TG, and NB; writing – original draft preparation: CO and NB; all authors reviewed and edited the manuscript.

## Conflict of interests

The patent application EP21174633 with the title ‘Computer assisted method for the evaluation of cardiac metabolism’ was filed by Charité—Universitätsmedizin Berlin as the employer of NB and Titus Kuehne, with both holding inventorship for this patent application. UK is a speaker of the Working Group Hypertension at the German Cardiac Society and a member of the German Hypertension League, Section Scientific Statements. The other authors declar no conflict of interest.

## Supporting information


**Table S1.** Pathway‐specific proteins with significant associations with metabolic capacities (separate file).

## Data Availability

The mass spectrometry proteomics data have been deposited to the ProteomeXchange Consortium via the PRIDE [63] partner repository with the dataset identifier PXD057532 and are publicly available as of the date of publication.
